# Two Worlds in One: What ‘Counts’ as Animal Advocacy for Veterinarians Working in UK Animal Research?

**DOI:** 10.3390/ani13050776

**Published:** 2023-02-21

**Authors:** Renelle McGlacken, Alistair Anderson, Pru Hobson-West

**Affiliations:** 1 School of Sociology and Social Policy, University of Nottingham, Nottingham NG7 2RD, UK; 2 School of Veterinary Medicine and Science, University of Nottingham, Leicestershire LE12 5RD, UK

**Keywords:** veterinary profession, advocacy, responsibility, laboratory science, animal research

## Abstract

**Simple Summary:**

The concept of advocacy is of increasing importance to the veterinary profession internationally, yet there are concerns about what it means in practice. This paper explores what ‘animal advocacy’ involves for veterinarians working in an area where its performance may appear particularly challenging, that of animal research. Based on an analysis of interviews with veterinarians working in UK animal research facilities, we aim to demonstrate both what ‘counts’ as veterinary animal advocacy in this domain and illustrate some of the tensions that may arise in its performance. Focusing on the themes of ‘mitigating suffering’, ‘speaking for’, and ‘driving change’ as three central ways in which veterinarians working in animal research facilities act as animal advocates, we draw out some of the complexities of advocacy for veterinarians working in areas where animal care and harm coexist. Finally, we conclude by calling for further empirical exploration of animal advocacy in other veterinary domains and for more critical attention to the wider social systems which produce the need for such advocacy.

**Abstract:**

The concept of advocacy is of increasing importance to the veterinary profession internationally. However, there are concerns around the ambiguity and complexity of acting as an advocate in practice. This paper explores what ‘animal advocacy’ involves for veterinarians working in the domain of animal research, where they are responsible for advising on health and welfare. In focusing on the identity of veterinarians working in an arena of particular contestation, this paper provides empirical insights into how veterinarians themselves perform their role as an ‘animal advocate’. Analysing interview data with 33 UK ‘Named Veterinary Surgeons’, this paper therefore examines what ‘counts’ as animal advocacy for veterinarians, considering the way their role as animal advocate is performed. Focusing on the themes of ‘mitigating suffering’, ‘speaking for’, and ‘driving change’ as three central ways in which veterinarians working in animal research facilities act as animal advocates, we draw out some of the complexities for veterinarians working in areas where animal care and harm coexist. Finally, we conclude by calling for further empirical exploration of animal advocacy in other veterinary domains and for more critical attention to the wider social systems which produce the need for such advocacy.

## 1. Introduction

### 1.1. Animal Advocacy and the Veterinary Profession

Acting as an ‘advocate’ for animals is framed as a key principle for the modern veterinary profession. For example, in their 2016 animal welfare strategy report entitled ‘Vets speaking up for animal welfare’, the British Veterinary Association (BVA) highlight the importance of advocacy to the veterinary profession, claiming that ‘Our opportunity to be advocates for animals may be the greatest of all and we have clear social, professional and legal responsibilities to do so’ [1] (p. 9). Also cited here is the World Organization for Animal Health’s assertion that veterinarians should provide leadership on ethical issues and ‘should be the leading advocates for the welfare of all animals’ (ibid), illustrating the global proliferation of the language of ‘advocacy’. Relatedly, in their ‘recalibration’ of the veterinary profession for the 2020s, De Paula Vieira and Anthony [2] argue that ‘society expects veterinarians to be animal advocates’ (p. 7), adding that ‘veterinarians will need to utilise their voice more’ when called upon to provide societal leadership on moral and welfare issues involving animals (ibid, p. 2).

Despite this shared language of veterinary ‘advocacy’, the concept of advocacy is complex and eludes a single definition, encompassing multiple, sometimes incongruent, values and necessitating different approaches in its practice. Capturing this ambiguity in their review of the theory and practice of advocacy in relation to social work, Forbat and Atkinson [3] (p. 322) observe that ‘At its simplest, advocacy means ‘speaking up’ for oneself or others. However, it is rarely that simple’. In the nursing profession, the nurse has long been theorised as a ‘patient advocate’, representing and intervening on behalf of those who are vulnerable [4]. Indeed, Gadow [5] (p. 81) put forward the influential concept of ‘existential advocacy’ as core to the nurse’s role, with nursing described as a process of participating with the individual’s unique experience of health and illness. For Gadow, existential advocacy is ‘based upon the principle that freedom of self-determination is the most valuable human right’ (ibid, p. 84).

Such humanistic conceptualisations of advocacy rely on principles of self-determination and ‘Promoting and protecting patients’ rights to be involved in decision-making and informed consent’ [6] (p. 35). These ideas are not easily transferable to non-human animal advocacy, given communication barriers between humans and animals. A possible parallel is paediatrics. Here, Waterston [7] (p. 155) summarises that ‘Paediatricians advocate for children because they are vulnerable and not usually able to speak for themselves’ and claims that such advocacy is importantly embedded in the principle of partnership with the family [8] (p. 587). However, this differs with animal patients, as Ashall, et al. [9] (p. 255) affirm that ‘Whilst medical consent protects a patient’s rights to make autonomous decisions concerning their own body, veterinary informed consent aims to protect an owner’s right to make autonomous decisions concerning their legal property’. This imperfect comparison illustrates the complexity of applying a concept such as advocacy across medical and veterinary contexts.

Indeed, existing veterinary literature confirms the challenges of advocacy in the veterinary context, including the question of balancing what the vet and the client (owner) may desire. For example, Morgan [10] (p. 116) distinguishes between the ‘animal advocate’ and the ‘client advocate’ professional model, with the former prioritising the interests of the animal and the latter prioritising those of the client, though they acknowledge that such models ‘do not have firm boundaries’ in practice. Others have utilised ideas of partnership similar to that raised by Waterston. For example, Gray and Fordyce [11] (p. 10) suggest that *both* the veterinarian and owner should act as advocates for the animal patient but add that this framework ‘requires elevation of the status of the animal patient to more than just ‘property’ or legal ‘object’’. Yet, if it is the case that the principle of autonomy has been applied to owners rather than animal patients, Hiestand [12] (p. 6) claims that ‘this misstep has serious consequences for both the integrity of the profession and animal patients’. While the ‘opportunity’ for veterinarians to be ‘advocates for animals’ may be great as the BVA urges, there is evident complexity for the individual professional in performing this advocacy on the ground.

In thinking about the current veterinary profession and the extent to which principles of advocacy inform the veterinary role, Main [13] has claimed that despite evidence of public trust in the veterinary profession, the profession needs to work harder to actually fulfil societal expectations regarding their advocacy role. Main adds that although clinicians are justified in their daily ambition to promote animal interests, ’a minimal scratch below the surface reveals obvious tensions in this well-intentioned mantra within the profession’. Furthermore, there is no ‘one’ singular veterinary profession, and particular challenges exist in different contexts. For example, in companion animal medicine, Kipperman and German [14] (p. 5) argue that failing to address problems such as obesity represents an abdication of their animal advocate role. Both Coghlan [15] and Hernandez et al. [16] promote a strong and active approach to patient advocacy in the veterinary profession. However, as the latter indicates, ‘Veterinarians embedded within certain animal production industries may find it particularly challenging to separate their ethical obligations to animals from their professional responsibilities to the corporation within which they are employed’ [16] (p. 6).

Another domain in which veterinarians are tasked with managing multiple responsibilities which may challenge their ethical obligations to animals is in animal research. In the UK, ‘Named Veterinary Surgeons’ (henceforth ‘NVSs’) advise on the welfare of animals kept at scientific establishments. As will be discussed, these veterinarians are involved in a contested practice in which animal care often coalesces with harm [17]. Attending to how such veterinarians perform their role through the ‘animal advocate’ identity stands to offer important empirical insights into some of its key characteristics, complexities, and limits in practice.

### 1.2. Animal Research, the NVS, and Animal Advocacy

In the UK, the presence of a veterinarian in all research facilities using animal models is mandated by law under the Animals (Scientific Procedures) Act 1986—‘ASPA’—which governs UK scientific animal use [18]. Being ‘nominated by the establishment license holder and specified in the establishment license’, the NVS is ‘responsible for, monitors and provides advice on the health, welfare and treatment of animals, and should help the establishment license holder to fulfil his/her responsibilities’ [19] (p. 150).

Working under the ASPA, the NVS is also required to uphold the principles of the 3Rs (Russell and Burch 1959). This requires that researchers must aim for ‘the replacement of animals with alternative mechanisms, where possible; the reduction of the number of animals required for a given procedure through statistical or other improvements; and the refinement of experimental procedures to minimise suffering and improve animal welfare’ [20] (pp. 605–606). Crucially, in addition to these core requirements under the ASPA, the NVS is also governed by their professional accreditation and have ‘professional responsibilities to the animals under their care, to other veterinary surgeons, to the public, and to the Royal College of Veterinary Surgeons (RCVS) (under the VSA (Veterinary Surgeons Act 1966))’ [21] (p. 72). This means that the NVS must ‘actively navigate the boundary between these two pieces of legislation, exercising professional judgement to reconcile potentially conflicting tensions arising from multiple professional accountabilities within the laboratory’ [22].

Existing empirical literature on the role of the NVS is sparse, but has focused, for example, on their route into the role from clinical practice [23]; their relationship with the 3Rs [24,25,26]; geographical dimensions of their ethical boundary work [27] and their role in promoting standardisation and regularisation [28,29]. Historians have argued that animal welfare groups were the first to propose the role, imagining the veterinarian to act as ‘the animal’s friend’ [30] (pp. 117–118). Today, the NVS is claimed to be an ‘advocate of the animal’ [31] (p. 26) and, indeed, acting as an advocate is described as the role’s ‘principal reward’ [32] (p. ii). However, existing literature also highlights some of the complexities of performing this advocacy role in practice. For example, Brouwer-Ince [32] (p. ii) has neatly summarised some of these challenges, which involve:

‘Challenges to your professional judgement, because your decisions may not be popular with some researchers; challenges to your scientific judgement where the broad scientific training of a veterinary surgeon may exceed the knowledge of a researcher in a narrow specialism; challenges to your personal ethics in making cost/benefit assessments if you feel that the scientific benefits are sometimes being overstated by researchers […]; challenges to your ability to give truly impartial advice if that advice costs your employer time and money; challenges to your personal integrity when researchers recognise that your views might carry more weight than theirs; and challenges to your personal social life because involvement in animal research still carries a stigma in some quarters.’

This list of challenges is evidence of the ethical complexity of the NVS role, with multiple factors complicating the veterinarian’s responsibilities for the welfare of research animals. In short, the NVS must balance multiple care obligations: caring for research animals, with the welfare of each individual being interwoven with the cohort, caring for the specific and broader scientific goals, and caring for patients and publics invested in both the expected scientific outputs and the protection of animal welfare.

Given these complexities, it is arguably unsurprising that some critics have voiced concern about the extent to which the veterinary profession should even be involved in the practice of animal research. Indeed, during the formalisation of the NVS role through A(SP)A (1986), there was some dissensus within the veterinary profession. As historians Kirk and Myelnikov [30] (pp. 117–118) observe, although the establishment of a veterinarian in the animal research domain was viewed as a compromise between welfare and scientific interests, the prioritisation of the veterinarian’s ‘duty of care for the animal made experiments a difficult sell to many veterinarians’. This is part of a critical debate beyond animal research, which continues today, about whether and how veterinarians are complicit in profiting from the bodily labours of animals [33], their commodification in both life [34] and afterlife [35], and whether or not the profession itself is compatible with ideas of animal advocacy [36].

With this wider context in mind, it becomes increasingly urgent to understand more about how the identity of the veterinarian as an animal advocate is performed by those working within the complex practice of animal research, in which animal care coincides and converges with deliberate harm. In this paper, we therefore examine the performance of veterinary advocacy within animal research facilities through analysis of interviews with NVSs. Our analytical approach draws on the literature already discussed in both the human and veterinary fields. In particular, being inspired by Friese [25], who, following Despret, asks ‘what counts’ to animal technicians ‘in the doing of their work’, we are similarly keen to explore what ‘counts’ as animal advocacy for NVSs. More specifically, we anticipate that the insertion of a veterinarian into the animal research facility as an animal advocate creates the potential for dissensus, establishing ‘the presence of two worlds in one’, to briefly borrow terminology from the political philosophy of Rancière [37] (p. 43), in which the logics of veterinary advocacy within the laboratory to ‘count’ the animals in one way exists alongside the logics of science, which include the requirement to ‘count’ the animals in another.

## 2. Methods

This study focusing on NVSs is part of a broader constellation of work under the Animal Research Nexus Programme (Animal Research Nexus Programme 2019; Davies et al. 2020). The aim of this specific study was to understand more about the role of veterinarians as key and underexplored actors in animal research facilities. Ethical approval for data collection was granted by the School of Veterinary Medicine and Science at the University of Nottingham (approval number 1800160608), and data collection took place in 2018. An interview guide was developed and discussed with an expert advisory panel of three NVSs and was trialled during two pilot interviews with NVSs. The interview format and question focus were subject to revision as the data collection progressed. NVS participants were contacted through snowball sampling initiated via personal networks and a call out during a specialist conference. Thirty-three interviews were carried out in person at a location chosen by the participant or over the telephone. Interviews were transcribed by a third-party under a confidentiality agreement. Transcripts were anonymised and decontextualised and each transcript was assigned a random but gender-specific pseudonym beginning with the letters M, N, O, or P.

An initial inductive thematic analysis [38] of the transcripts was originally undertaken by the second author, using the qualitative data analysis software NVivo 12. This aimed to create analytic themes that reflect meaning-based patterns across the interview dataset. The use of this inductive approach was particularly important as NVSs are an understudied group, and there was minimal prior research on this group to draw upon to inform the study and analysis. Through two coding cycles, this phase of analysis concentrated on drawing out participants’ physical and conceptual actions using their own phrasing and language to prioritise their voices in driving the analysis [39] and then worked to broaden and deepen these categories, focusing on the patterns underpinning them [40]. This coding process was not linear, and in developing the second cycle of codes, the first cycle categories were sometimes modified in title or content as the analyst clarified the key themes.

Following the initial phase of coding, a second phase of thematic analysis was undertaken by the first author. Working with the second author’s coding structure in NVivo 12, the first author created subcodes within the thematic category of ‘Advocate for the animals’. With animal advocacy already identified as an important theme in the dataset, this round of coding was guided by a broad questioning of what ‘animal advocacy’ means to Named Veterinary Surgeons. In doing so, the first author sought to examine how the advocate identity might be configured through particular orientations, enactments, and understandings of their role. These issues formed the basis of discussions between the authors. In drawing on this analysis, this paper therefore explores how the enactment of animal advocacy was characterised by Named Veterinary Surgeons, focusing on the three key aspects of mitigating suffering; speaking for; and driving change that were prominent throughout many of the interviews. Though overlapping in many ways, these themes are organised separately for clarity.

This analysis aims to elucidate how veterinarians working in the ethically complex and contested arena of animal research perform ‘advocacy’ and draw out the tensions that may exist within this. Such tensions have the potential to complicate the dominant image of the veterinarian as ‘the kindly, trained person who knows how to take care of [one’s] companion animal’ [41] (p. 69). In other words, focusing on how veterinarians frame their advocacy for research animals within complex structures in which care and harm coalesce [17] may offer important insights into the reality of the veterinary profession, which is ‘considerably more murky’ than the dominant image [41] (p. 69). As will be returned to in the discussion, such analysis may reveal the limits of veterinary animal advocacy in the research context, how it might be expanded, better supported, and what can be expected of Named Veterinarians as advocates for research animals.

## 3. Results

### 3.1. Mitigating Suffering

#### 3.1.1. Preventing and Limiting ‘Unnecessary’ Suffering

A key way in which participants described enacting their role as an ‘animal advocate’ was through preventing and limiting suffering via the safeguarding of animal welfare. This may sound simple initially, but the analysis reveals how complex this can be in practice. As the following examples illustrate, this can mean drawing lines between ‘necessary’ and ‘unnecessary’ suffering [42,43,44]:

‘So, whatever is in the best interest of animals here. Like, say you have the animals occasionally suffer and you have to distinguish between the scientific benefits for study, and animals shouldn’t suffer. And it depends on type of the study […] you have to really be aware of these things and you have to stop everything, even despite millions of pounds invested and you have to say, ‘Look…’’

(Obadiah).

‘Really just to prevent pain, suffering, distress and lasting harm or if you can’t prevent it and obviously, if you’ve got justification and you’re allowed to carry it out, that’s what it’s all about. But to mitigate it as much as possible and so I would say it entirely centres around how we control pain, suffering, distress and lasting harm […] I mean the lasting harm is a bit difficult but the pain, suffering and distress’

(Owen).

As both participants indicate, some amount of animal suffering may be an accepted part of a study and so the NVS must work, as Owen states, ‘*to mitigate it as much as possible*’. However, at certain points, the NVS may call for an end to an animal’s use within a study, meaning the possible disruption of research aims, as Obadiah notes ‘*even despite millions of pounds invested and you have to say, ‘Look…*’’. To give another example, the extract below from Mia’s interview demonstrates their desire to ensure that the animals are ‘*looked after properly*’ and that research does not go ‘*too far*’ in compromising animal welfare. This is also linked, however, with apparent acceptance of the value of animal research:

‘[…] my role is there to protect them and to stand up for them, so that’s how I, presumably, in my own mind, justify it to myself, that I am involved in research in that way. Partly because I see that there’s a benefit in the long run but because I feel I’m there to stand up and make sure that those animals are looked after properly and appropriately and that research isn’t allowed to go too far’

(Mia).

Through their focus on animal welfare, NVSs can therefore be seen to articulate the importance of preventing and limiting ‘unnecessary’ suffering to their performance of the animal advocate role. However, the analysis reveals that there are limits to this in practice and that, sometimes, the NVSs’ animal advocacy may require ‘ending’ suffering.

#### 3.1.2. Ending Suffering

In some cases, preventing or minimising suffering is not deemed possible or sufficient and thus NVSs report the use of ‘euthanasia’ to end the suffering of particular animals. For instance, Oliver indicates that if measures to make animals more comfortable are not working then, as in general practice, the NVS will recommend euthanasia:

‘[…] it is the NVS job, to be the animal’s advocate and if we feel that the animal’s uncomfortable we will always recommend euthanasia but then that’s the same as in general practice. I think where the problem is sometimes I think, I’ll see an animal and I’ll think well this might get better if I do this or do that. And two days later it’s still not much better and you think well do we carry on? Because Home Office guidelines would almost certainly say no, you stop at this point’

(Oliver).

In animal research, such decision making reflects the guidance against using death as an endpoint for an animal’s use in a study. Rather, researchers are encouraged to implement ‘humane endpoints’. This involves defining ‘clear, predictable and irreversible criteria which substitute for more severe experimental outcomes such as advanced pathology or death’, and, when these are reached, the animal is humanely killed [45].

However, decision-making around euthanasia or humane killing can involve more than assessments of individual animal welfare when deciding what counts as ‘too much’ suffering. In general veterinary practice, it may be complicated by the human–animal bond [46,47], the client’s judgment of the animal’s health [48], their wider interests, and their finances [49]. In animal research, with the ‘two worlds’ of veterinary professionalism and scientific practice existing ‘in one’, decisions around killing an animal may also be complicated by the competing interests of researchers and other institutional actors, obligations towards the research aims and helping to ensure meaningful outputs from the experiences of animals, and the statistical value of each animal which interweaves their individual welfare. Indeed, as Michelle discusses, this can create areas of conflict:

‘I think you can have areas of conflict where the researchers say, ‘This is a very valuable animal and …’, it always happens, whenever anything, there’s a problem with the animal, it always becomes the most valuable animal they have in their research and they want to continue using it to get the results they want. And then you can sometimes have conflict with them in that you’re saying, ‘No, I’m sorry, you have to finish, it has to go’, particularly if they are a researcher who’s got a very big grant and is basically going to run to the Vice Chancellor at the university and take the attitude, ‘You’re trying to stop my research’. So, you can get into situations like that and you basically, I think you have to stand up for the animal’

(Michelle).

In describing a situation in which the NVS’s call to remove or *dis*count an animal from a study may meet challenge from researchers, Michelle states that ‘*you have to stand up for the animal*’. In this example, this can mean ending their life. This extract raises difficult questions, discussed in the wider literature, about the extent to which euthanasia should be understood as a ‘gift’ [50], or whether it should be identified as a harm [51,52,53]. For example, Coghlan [15] (pp. 360–361) contends that ‘The denial that animals can be harmed by death is often connected with a quasi-technical notion of animal ‘welfare’ and ‘Since veterinarians have power over animal life and death, this denial is significant’. In the animal research context, one could also question whether the relative absence of opportunities for rehoming research animals ([54,55]) could be seen to reinforce decisions around humane killing. In other words, the wider context of animal research needs to be appreciated, within which the advocacy of NVSs is articulated.

Overall, we have demonstrated that the NVS’s work to prevent, limit, and end animal suffering is an important part of their advocacy. However, this is arguably premised on a professional acceptance of ‘necessary’ suffering. Critics have argued that such a conceptualisation of ‘necessary’ suffering is itself morally flawed. For instance, Fox [44] (p. 30) has argued that ‘if animals’ lives have value independent of their interests to others, all of their suffering is morally unjustified’. Likewise, in challenging the ‘necessity’ of scientific animal use more broadly, Francione [43] (p. 248) asserts that, although ‘problematic in a number of respects’, the ‘use of nonhumans in biomedical research may involve a plausible claim of necessity […] But such a claim, even if justified, cannot serve to provide a satisfactory moral basis for this use of animals’. Such contestation around the premise of ‘necessary’ suffering which is seen to inform how the welfare of research animals is conceived therefore highlights tensions within the identity of the NVS as an animal advocate within the worlds of scientific practice.

### 3.2. Speaking for

#### 3.2.1. Speaking for Research Animals

As well as constructing the performance of advocacy for research animals as intervening in suffering, the NVSs interviewed also emphasised the representational dimensions of their role, describing their work to ‘speak for’ the animals and act on behalf of their best interests. As Margaret discussed:

‘I guess what I do is, it’s really just to try and speak for them, be an advocate for the animals and safeguard them. Most researchers are not intending to be cruel to the animals, it’s more sometimes they just don’t understand them well enough to know the consequences of what will happen for the animal if they do something, or they don’t see the signs which can be quite subtle, particularly if you’re dealing with mice or sheep or rats or prey type animals, they can look at an animal and they won’t see what I will see or the technician would see, that’s not intentional, it’s just they don’t know. So, it’s really being there for the animal and taking care of them’

(Margaret).

This ‘speaking for’ is here intended to safeguard animals from the harms of the research and the researchers. Margaret clarifies that the harms posed by the latter are not intentional but rather result from a lack of certain knowledge. Indeed, as she puts it, researchers ‘*can look at an animal and they won’t see what I will see or the technician would see*’. With their embodied expertise and the identification of welfare impacts and diagnosis of illness and disease this enables, Margaret’s description portrays the NVS as thus able to count the animals as part of an ‘intervention of the visible and sayable’ [37] (p. 45). This means representing the interests of research animals, speaking up for them on issues that may be missed by researchers who lack the same levels of knowledge of or familiarity with the presentation of animal suffering.

Similarly framing their advocacy for research animals as juxtaposed to the interests of researchers, Owen describes how they are responsible for speaking up for the research animals ‘against’ the positions of the researchers. In this extract, the initial ‘NACWO’ refers to the Named Animal Care and Welfare Officer:

‘The Home Office love to call ASPA “a caring Act” because there were two care people in there, the NACWO and the NVS and we are named care people, and I see my job as being a care person to speak up on behalf of the animals against the wicked scientists who are wanting to do all sorts of naughty things to them […] it is what I feel, we are. So when I’m called in, in a dispute situation, the dispute is always between the NACWO and the scientist and it’s always that the NACWO is saying, ‘I think this animal ought to be put down’ and the scientist saying, ‘can’t we hang on a bit longer?’’

(Owen).

Defining the NVS role as a ‘*care person*’, Owen here identifies their role and that of the NACWO as posing a challenge to the scientists, whose interests concern the animal’s use as an experimental model. Perhaps half-joking in their description of the moral character and interests of scientists, portraying an oppositional relationship between an animal-loving veterinary surgeon [56] and ‘*wicked scientists*’, Owen illustrates the conflict that can be expected as part of their role. Yet, later in the interview, Owen discusses how their advocacy can be enacted in conjunction *with* rather than *against* scientists if a working relationship has been built from the project’s nascent stages:

‘[…] but if you’ve met them before their application goes in and you’ve talked to them and you’ve advised them and they’ve explained what they’re trying to do and you’ve explained how they can best do it in your opinion, hopefully you’re all on the same side and I think in the best units, that’s what’s happened, so there isn’t much of a conflict’

(Owen).

Owen’s claim here is that it is good practice in regard to animal welfare if the NVS is involved in a project from its inception. In this case, the NVS’s’ advocacy can take the form of sharing expertise, with the NVS *advising* how best the research aims can be met whilst in-keeping with animal welfare and meaning that all actors are ‘*on the same side*’. Other participants described how an embracing of the 3Rs by scientists can have a significant impact in changing the NVS’s role from one of opposition to one of collaboration.

Relatedly, in representing and speaking up for the interests of research animals, some participants framed independence as a crucial part of the NVS role, as Nadir expresses:

‘What I hope I achieve here is to be seen just as an honest broker, someone who can be relied upon to say what they think, and to not carry any political baggage. My job is to represent the interests of the animals as best I can in a complicated structure where the interests of the animals are one part of a larger programme that the institute or the company is trying to achieve’

(Nadir).

With the NVS’s responsibility to speak for research animals often necessitating challenging the decisions and interests of others, their independence from the interests of researchers and the institution is significant for maintaining appropriate levels of impartiality. However, as Nadir implies, such independence relates not only to the research infrastructure, but also wider political agendas. In being seen as uninvested not only in the research outcomes and institutional goals but also in relation to other stakeholder agendas, Nadir discusses how they aim to present their role to others in the facility as an ‘*honest broker*’.

Interestingly, in Pielke’s [57] influential typology of the roles that scientists may play in political issues, the ‘honest broker’ is distinct from the role of ‘issue advocate’. Indicating the key difference between the two, Pielke observes that the broker seeks to ‘expand (or at least clarify) the scope of choice’ whilst the advocate ‘seeks to reduce the scope of available choice’ (ibid, p. 18). In advocating on behalf of the research animals’ interests, the NVS may be seen as carrying ‘*political baggage*’ of a kind. However, Nadir’s excerpt suggests that, to perform their role effectively, it is important to be perceived as helping and not hindering researchers, providing impartial insights into how animals can be used and counted within welfare parameters instead of working to *dis*count or limit their use altogether.

#### 3.2.2. Speaking for Society

The advocacy element of the NVS role may be framed, not only as representing the interests of research animals within a facility, but also representing wider society’s interests in animal welfare. As Nicholas suggests:

‘[…] The other aspect which sometimes gets lost is that the NVS is the people’s representative of the animals, they’re there to be on the side of the animals and the role was created to put an independent person into the facility. And one of my friends, just coincidentally, was part of the campaigning around the ’86 Act, he said one of the things we were really pleased about was that we got the vets in there because they wanted someone on the side of the animals in the facility, and I think that’s probably the area that we need to make sure is still understood’

(Nicholas).

As well as caring for the research animals in their charge, Nicholas indicates that an important responsibility of the NVS is to act as ‘*the people’s representative of the animals*’, representing the wider public interest in the protection of animal welfare. This perspective emphasises that the NVS is more than ‘just’ a representative for the animal, they also perform a social role mediating between science and society. This reflects the importance of public opinion in shaping the governance of animal research, a topic that has been itself explored in some depth [58,59,60,61]. In this case, public concern for animal welfare and trust in the veterinarian to embody ‘*the side of the animals*’ is considered to have created a professional niche for veterinary surgeons in the establishment of the NVS role.

Other participants invoked wider publics in their claims about their complex professional responsibilities:

‘At the end if the animal is not fine, that research is not going to be valuable. So, I see that as a double responsibility. I’ve got the responsibility for the animal and the animal needs to be fine, so it should be done properly on the animal and everything. Because otherwise those days are not worthy, if those days are not worthy, I’ve wasted those animals. At the same time, what I want as a taxpayer or whoever will benefit one day, I’ve also wasted the money, time, effort. So, research at the end comes from money, that comes from people via tax, via charities, whatever you want that believes in that, and they want to make a change for the future’

(Nathalie).

Here, publics are figuratively present in the form of taxpayers who contribute to the funding of scientific research and as patients who are expected to ultimately benefit from the research. In this framing, the NVS is constructed as responsible for assisting the return on this financial and affective investment by ensuring that animal research is conducted to appropriate standards that facilitate the materialisation of promised and expected outputs. Publics are thus enrolled and counted here as one of the ‘objects of care’ [62] (p. 60) for the veterinarian, alongside the animals and the scientific output.

In summary, NVSs in our study articulate obligations for multiple objects of care, with their relationship to animal welfare being entangled with the project’s scientific aims and also the wider interests of publics invested in both the research outputs and the protection of animals. Indeed, Ashall and Hobson-West [63] (p. 292) argue that ‘NVS responsibilities to a scientific establishment under A(SP)A should be viewed as additional to the multiple responsibilities which are faced by all veterinary surgeons, and which have previously been identified as a potential source of ethical conflict’. The plurality of the NVS’s care obligations suggests that although their independence from the research itself is important, they must negotiate logics that count the animal as an experimental model, shaping the NVS’s advocacy for research animals.

### 3.3. Driving Change

#### 3.3.1. Changing from the ‘Inside’

Thus far, this analysis has highlighted the tensions involved in the NVS’s performance of the animal advocate role. Given the limits of advocacy in the scientific context within which NVSs operate, it is perhaps understandable that some participants connected their role as an animal advocate to driving change in research practice. Indeed, prompted by the interviewer’s discussion of the potential conflict in constructing the NVS as an animal advocate whilst their work can be seen as facilitating uses of animals that contravene their interests, Margaret argues that by being on the ‘inside’, the NVS role provides an opportunity to affect change:

‘One of the reasons I sort of got involved in it because with research, it is a difficult thing but I think if you’re going to actually make a difference and influence anything, you’ve got to be in amongst it, it’s no good kind of standing outside and shouting at the walls, you’ve got to be there to see what happens and understand what’s happened and try and influence it. And that’s partly why I’ve got involved with it and partly why I’ve taken up some difficult conversations with some researchers and the Dean […] I’m asking ‘why?’, but you’ve got to have that conversation because if you don’t, you’re failing really because you’ve got to point it out because otherwise nothing will change’

(Margaret).

The NVS here is framed as an agent of change within animal research facilities, able to initiate difficult conversations with scientists and organisational actors which would not be possible if they were ‘*standing outside*’. This was contextualised differently by Nadir, who contrasted professional responsibilities towards animals with ethical responsibilities to advancing human medicine and preventing human illness and deaths. Nadir listed multiple relatives and members of their local community who had died of various diseases or lived with chronic conditions, and having justified animal research in this context echoed Margaret’s sentiment:

‘I have a foot in both camps. Although my professional responsibilities are to the animals I look after, I also have moral and ethical responsibilities to my species, particularly to my own family and friends and relatives, many of whom have struggled with the sorts of diseases which the use of animals here is trying to address. So there is that conflict going on all the time. […] That’s where animals come into the research picture. However reluctantly they appear on the horizon, they are there, and once you accept the fact that research is probably justified, then I’d rather be involved in it, doing it as well as I can, than standing on the outside griping’

(Nadir).

Nadir draws a clear distinction here between human and non-human animals, having a ‘*foot in both camps*’ that contextualises their ethical and professional responsibilities towards the two groups. However, following Clarke and Knights [64] (p. 269), this assumption that animals are needed to address historic failures in human medicine is to reproduce narratives of ‘anthropocentric masculinities’ which are common in veterinary practice, whereby ‘men seek to transform animals and nature into orderly, predictable and serviceable objects of human(istic) desire’. Interesting here, is the way in which, Nadir works through the tension they articulate between their ethical and professional responsibilities by being on the ‘inside’ of animal research. Such justifications of the NVS role and the possibilities they provide to advocate for research animals, rather than ‘*standing on the outside griping*’, raise broader questions around the lack of opportunities for both publics and professionals outside of organisational frameworks to participate in decision-making processes and influence change within animal research.

#### 3.3.2. Changing Practice

In discussing their desire to affect change, several participants referred to the implementation of the 3Rs as a key responsibility of the NVS. In working to implement the 3Rs, the NVS’ animal advocacy is framed as necessitating an approach which goes beyond individual animal welfare and is also concerned with driving best practice. As Parker discussed:

‘Yeah, because your main job as a vet, you are there for the animals, but the reason you are employed by the institute is not only to tick boxes, so you are there to provide, basically you have to justify your salary. So, you’ve got to try to be an [inaudible 64:49], a 3R Hercules. OK, so you’ve got to try to stimulate refinement all the time, in the husbandry, on the experiments, and then bring people to use more reduction, and the replacement is very difficult as an NVS because it’s really, your mind is about the animals so replacement is very difficult’

(Parker).

Parker differentiates between the core work of a veterinarian who is ‘*there for the animals*’ and the work of the NVS who is ‘*employed by the institute* […] *not only to tick boxes*’, describing the latter as going further than caring for animal health and welfare and also working to drive uptake of 3Rs approaches and techniques. In particular, Parker characterises their work around replacement as especially difficult, with their role embedded in the day-to-day animal *use*.

Similarly, other participants highlighted the important yet tricky nature of the NVS’s advocacy for the replacement of animal models. As Maeve describes in this interview exchange:

‘I still see myself as an advocate for the animal. I think as a professional, well I’m maybe more outspoken than others but I’m not pro animal research, I never was but professionally, I can live with rules and if the product licence says this is what they’re allowed to do, I’m fine with that. I think we should be much more involvement in replacement, which we aren’t because…’

Interviewer: NVSs should?

Yes, we should understand it, we push it away from us because it’s complex and we don’t have time to look into this and also, I’m still seeing myself as an advocate. I give independent advice, this is what I do, I should be there to question, that doesn’t mean I should make life difficult because I don’t like somebody’s notes, that is completely different from giving independent advice in a welfare situation but I will be the first one who, if somebody is hammering on about needs to do single housing, I’ll be saying, “If you need to do single housing and you do surgeries, you need to single house earlier so that once stress is over and then do the surgery and then you do this”, and a lot is to teach people to think about what they don’t think about’

(Maeve).

In this excerpt, Maeve makes a distinction between ‘*giving independent advice in a welfare situation*’ and challenging decisions which may impact on animal welfare (e.g., single housing of animals). Such a differentiation suggests that for the NVS to be effective in their animal advocacy, they must recognise the appropriate moments when challenge is called for and appreciate the equal importance of advice and education. Indeed, in the context of paediatric medicine, Waterston [7] (p. 156) states that ‘Advocacy does not mean storming the White House or number 10 Downing Street; instead, it is using the wherewithal of professional expertise, credibility and experience to draw attention to issues and execute beneficial changes’.

Yet, the specific challenges that surround the NVS’s work in the area of replacement mean that the ways they can advocate is more easily directed at improving the ways in which animals are used (in terms of getting the best scientific output from the animals and safeguarding animal welfare) rather than preventing their use at all. Such barriers to advocating for replacement may hint at the pitfalls of becoming professionalised *within* systems one may aim to ultimately transform or disrupt. Hence, as some extracts included earlier in this section suggest, although being on the ‘inside’ may be said to enable veterinarians to question scientists on their animal use and hold challenging but important conversations aimed at stimulating better practice, the orientation of the NVS role *towards* animal use may impede their advocacy around the ways in which animals might be replaced.

## 4. Discussion

In seeking to enrich understandings of what ‘animal advocacy’ means for veterinarians working in animal research, this paper has focused on three key ways in which Named Veterinary Surgeons enact their role as an ‘animal advocate’, that is, through mitigating suffering, speaking for, and driving change.

The first thematic section analysed the link between the NVS’s advocacy and their work to mitigate animal suffering and considered how the requirements of the research context may orient the advocacy of NVSs towards protecting animals from the impacts of a study, rather than cultivating lives ‘worth living’ [65] per se. This section also highlighted how the mobilisation of distinctions between necessary and unnecessary involves some acceptance of degrees of animal suffering; in other words, NVSs bring together logics of veterinary professionalism and scientific practice. Finally, this section touched on how the work of NVSs to limit unnecessary suffering plays out within the confines of the research infrastructure when an animal’s suffering cannot be controlled, with euthanasia or ‘humane killing’ discussed by several participants as an important part of how their advocacy is practiced. Given existing challenges around the rehoming of research animals [54,55], advocating for the humane killing of animals may often be one of the only options available to NVSs when advocating for the removal of an animal from a study.

Scholars are increasingly recognising the way in which care, harm, and killing coalesce in animal research [17,66] and in wider areas of veterinary practice. Indeed, Venkat [67] (p. 1) considers that cruelty ‘might be figured as an unavoidable aspect of the relation of dependency between animals and their human caretakers’ and suggests that in and through veterinary medicine ‘humans and their particular forms of cruelty are perhaps unavoidably implicated in the deaths of animals’ (ibid, p. 14). This reminds us that the case of veterinary involvement in animal research is not the only example of how veterinary animal advocacy may be conflicted or contested. Our analysis thus might contribute to these wider debates by serving to highlight the ways in which veterinary animal advocacy is inherently complicated and open to contestation and involves a negotiation of multiple and often divergent interests within broader paradigms of anthropocentricism.

The second thematic section, that of ‘speaking for’ research animals and wider society, illustrated how many NVSs framed their relationship with researchers as often being one of challenge, with the professional dynamics of their two different worlds associated with a common occurrence of conflict when they exist together. However, some participants described how when the NVS’s involvement from the formation of a study and the prioritisation of animal welfare and the 3Rs is evident throughout, then the NVS’s advocacy can more often take the form of *advising* rather than *challenging* researchers. Although the participation of the NVS in their institution’s Animal Welfare and Ethical Review Body (AWERB) is mandatory in the UK, this analysis suggests the importance of relational dimensions of cooperation, beyond the formal institutional processes of ethical review. However, we also considered how presenting themselves as an impartial mediator may stir tensions with their identification as animal advocates, with their advocacy framed here as *assisting* with the scientific use of animals within acceptable welfare parameters, rather than advocating for their interests outside of this paradigm.

This last point raises questions beyond the identity of veterinarians and encourages us to consider which versions of the *animal* are being counted or represented within animal advocacy. Here we find Weich and Grimm’s [68] analysis useful in arguing for more ‘critical scrutiny of the normative social structure’ in which an animal patient’s interests emerge and an ‘interrogation of animals’ activities within that structure’. With understandings of research animals fluctuating between the naturalistic and the analytical [69], model and pet [70,71], individual and colony, wild and domestic, this provocation pushes us to consider what *kinds* of patients are produced through animal research for which the veterinarian is responsible. Future research might therefore focus more directly on how Named Veterinarians and other professionals who advocate for the welfare of research animals construct their animal patients, the meanings they make of their health and illness, what these involve, and where they begin and end. Further work could also explore where this differs internationally (as called for by Anderson and Hobson-West [27]). Future studies might therefore also question the extent to which animal advocacy in the research context is complicated by conceptualisations of the animal as both patient and scientific tool, and how their other roles and accordant interests may or may not be given opportunities to flourish in research facilities.

The final theme explored the way in which NVSs discuss advocacy as partly about driving change in practice. That some participants described the NVS role as enabling them to advocate for research animals by permitting them entry *inside* the research infrastructure arguably draws attention to the current lack of opportunity for veterinary professionals outside of animal research to participate in driving good practice and inform policymaking on scientific animal use. This point relates to broader calls to open up this arena to more actors, including publics [61]. The current paper also highlights how the construction of the role of the NVS as an ‘independent insider’ might generate both opportunities and limitations for veterinary animal advocacy for research animals. For example, this analysis has pointed to the ways in which a focus on the day-to-day *use* of animals may restrict the NVS’s animal advocacy around the *replacement* of animals. Given the relationship between the NVS and wider publics discussed in the first analytical section, and evidence that publics are invested in full replacement of animals with alternative models [72,73], this may prove problematic in the longer term.

## 5. Conclusions

This paper began by demonstrating the way in which the veterinary identity is closely associated with acting as an animal advocate. However, in doing so, we have also argued that definitions of advocacy are problematic and illustrated that transferring the concept from the human to the animal patient is not straightforward. We then introduced the role of the NVS and the ways in which assumptions about advocacy are built into this role. Whilst the role of the NVS is quite specific, we hope that this analysis will serve to enrich the definition of ‘animal advocacy’ in veterinary practice more broadly. Indeed, as Anderson and Hobson-West [23] (p. 6) have previously argued, ‘Focusing on the experiences of Named Veterinarians may therefore help us grapple with some of these fundamental questions about the future of the profession and the role of veterinary expertise in society’. This is arguably particularly urgent given the veterinary profession finds itself at ‘somewhat of a crossroads’ regarding the nature of its professionalism and the future of the profession in the UK as it addresses a number of concurrent crises [23].

Returning to the BVA’s proclamation that veterinarians’ ‘opportunity to be advocates for animals may be the greatest of all and we have clear social, professional and legal responsibilities to do so’ [1] (p. 9), this paper has illustrated the trickiness in defining veterinary advocacy for animals, demonstrating the tensions surrounding the veterinarian’s advocacy for animals whilst working within arenas governed by both care and harm. This description not only applies to the animal research context, but arguably pertains to the majority of human–animal relations and the veterinarian’s work as a, not independent, but thoroughly partial mediator of them [74]. After all, veterinarians are not external to the cultures which radically shape their patients’ experiences of health and illness. Given this broader context, we would encourage more practical support for veterinarians working in all fields, to scrutinise the specific structures and practices of power which produce animals as patients in the first place.

This argument has implications for the social scientific study of veterinarians more broadly, signalling the need for further research which focuses, not just on the experiences or perspectives of veterinarians, or the interactions between veterinarian and client, but which critically examines the relationship between the veterinary profession and wider society. This in turn would demand further exploration of the kinds of veterinary patients that come into being through human–animal relations and allow consideration of the extent to which the animal as *more-than-patient* might be included in veterinary animal advocacy.

## Data Availability

The dataset on which this paper draws has been anonymised and deposited in the UK Data Archive, subject to an embargo: Davies, G., Greenhough, B., Hobson-West, P, Kirk, R., and Roe, E. (2033) Animal Research in the UK, 2017–2023. UK Data Service. SN: 8942, DOI:10.5255/UKDA-SN-8942-1.
